# Neutrophil responses to RSV infection show differences between infant and adult neutrophils

**DOI:** 10.1136/thorax-2023-220081

**Published:** 2023-12-02

**Authors:** Elisabeth Robinson, Shyam Sawhney, Mario Cortina-Borja, Anna L David, Claire M Smith, Rosalind L Smyth

**Affiliations:** 1 UCL Great Ormond Street Institute of Child Health, UCL, London, UK; 2 School of Medicine, Imperial College London, London, UK; 3 UCL Elizabeth Garrett Anderson Institute of Women's Health, UCL, London, UK

**Keywords:** airway epithelium, innate immunity, neutrophil biology, paediatric lung disaese, respiratory infection

## Abstract

**Introduction:**

Respiratory syncytial virus (RSV) causes a severe respiratory condition, bronchiolitis, in infants but not in adults. Bronchiolitis is characterised by neutrophilic infiltration in the airways, but whether neutrophils enhance recovery from infection or contribute to its pathology remains unknown.

**Methods:**

We used a novel in-vitro model to compare term umbilical cord blood (infant) (n=17 donors) and adult neutrophils (n=15 donors) during migration across RSV-infected differentiated human nasal airway epithelial cells (AECs) in a basolateral to apical direction.

**Results:**

Greater numbers of infant neutrophils (mean (95% CI)) (336 684 (242 352 to 431 015)) migrated across RSV-infected AECs to the apical compartment (equivalent to the airway lumen) compared with adult neutrophils (56 586 (24 954 to 88 218)) (p<0.0001). Having reached the apical compartment of infected AECs, much greater numbers of infant neutrophils (140 787 (103 117 to 178 456)) became apoptotic compared with adult (5853 (444 to 11 261)) (p=0.002). Infant neutrophils displayed much greater expression of CD11b, CD64, neutrophil elastase (NE) and myeloperoxidase (MPO) than adult neutrophils at baseline and at all points of migration. However, as adult neutrophils migrated, expression of CD11b, CD64, NE and MPO became greater than at baseline.

**Discussion:**

The high proportion of infant neutrophils migrating across RSV-infected AECs correlates with the neutrophilic infiltrate seen in infants with severe RSV bronchiolitis, with large numbers undergoing apoptosis, which may represent a protective mechanism during infection. Compared with adult neutrophils, infant neutrophils already have high expression of surface markers before contact with AECs or migration, with less capacity to increase further in response to RSV infection or migration.

**WHAT IS ALREADY KNOWN ON THIS TOPIC**

- Respiratory syncytial virus (RSV) is the leading cause of bronchiolitis, a seasonal respiratory infection affecting primarily young children and babies but has also been shown to contribute to significant morbidity in elderly populations. Neutrophils have been shown to form the majority of airway cellular infiltrate during severe RSV infection, but whether this is beneficial in clearing infection or worsening clinical inflammation is poorly understood. This study explores in an in-vitro model the differences in neutrophilic migration, viability and expression markers to further understand this phenomenon.

**WHAT THIS STUDY ADDS**

- Term cord blood (infant) neutrophils have greater expression of activation markers before migration; adhere to and migrate across airway epithelium in greater numbers, after which they *undergo* apoptosis to a greater proportion than adult neutrophils. This suggests that there are intrinsic differences in the neutrophil response between adult and infant neutrophils which may account, at least in part for the severe condition of bronchiolitis seen in infants.

**HOW THIS STUDY MIGHT AFFECT RESEARCH, PRACTICE OR POLICY**

- Modulation of heightened recruitment of neutrophils may be of therapeutic benefit in the management of bronchiolitis.

## Introduction

Respiratory syncytial virus (RSV) is the predominant cause of lower respiratory tract infection (LRTI) and a leading infectious cause of hospitalisation and death in infants under 1.^1 2^ RSV circulates in seasonal epidemics, peaking in incidence over winter in the UK causing significant infectious burden in infants and the elderly.^3^ Clinically, RSV LRTI causes a spectrum of illness in infants ranging from a self-limiting illness to bronchiolitis requiring hospitalisation.^4^ Severe viral bronchiolitis in infancy is associated with development of viral induced wheeze and asthma in later childhood.^5 6^ There are no licensed vaccines for RSV infection, and current treatment guidelines recommend supportive care.^7 8^

In infants hospitalised with severe bronchiolitis, neutrophils are the predominant immune cell recruited to the airways, accounting for around 80% of the cellular infiltrate at peak of clinical illness.^9–11^ However, to what extent these neutrophils influence viral clearance and recovery or contribute to increased inflammation remains unclear. CD11b, an integrin essential for neutrophil migration, has been shown to be raised on blood and bronchoalveolar lavage (BAL) neutrophils of infants with RSV bronchiolitis; and CXCL8 (IL-8) (a potent neutrophil chemoattractant) concentration in has been shown to correlate with RSV bronchiolitis severity, suggesting neutrophils may contribute to viral pathogenesis.^12 13^ RSV mRNA transcripts have been recovered from neutrophils in both BAL and peripheral blood of infants with severe RSV, demonstrating neutrophils interact directly with RSV in infected infants.^9^ Neutrophil-derived products such as IL-9 and granule contents neutrophil elastase (NE) and myeloperoxidase (MPO) have also been shown to correlate with clinical disease severity.^12–14^ Using an in-vitro model of RSV-infected airway epithelial cells (AECs), our group has observed that increased neutrophil transepithelial migration through RSV-infected AECs is correlated to greater AEC damage, reduced ciliary beating and loss of AEC tight junctions.^15^

Adults do not suffer from the same clinical syndrome of RSV infection (bronchiolitis) seen in infants, and severe disease in adults is predominantly in immunosuppressed or elderly populations.^16^ This difference was thought to be due to an infants’ lung physiology with smaller airways being more susceptible to plugging by mucus and to the consequences of inflammation on gas exchange and airway clearance.^17 18^ More recent research suggests that clinical severity of infection is determined by neutrophil-mediated inflammation in both infants and adult human challenge models of RSV infection^19^; and so functional differences between adult and infant neutrophils may hold a key to differential severity and clinical syndrome.

Functional differences have previously been documented between infant compared with adult neutrophils, including reduced degranulation, neutrophil extracellular trap (NET)-osis and phagocytosis capacity, and greater neutrophil CXCL-8 production in response to Toll-Like Receptor stimulus.^20–22^ Neonatal neutrophil chemotaxis has been reported to be depressed in comparison to adult, with term infants achieving parity of chemotactic function in the days after birth.^23–25^ Apoptosis is also reported to be delayed in neonatal neutrophils compared with adults, which may be partially explained by relative lack of CASPASE 3 and contribute to prolonged inflammation.^26–28^ As it is not ethically justifiable to sample small healthy infants, to obtain sufficient neutrophils for these laboratory studies, as has been done previously,^21 23 29 30^ this study has used neutrophils derived from term umbilical cord blood as a biological surrogate for infant neutrophils.

The aim of this study is to compare the ability of healthy infant and adult neutrophils to migrate across RSV-infected human AECs and to measure differences in neutrophil viability and key activation molecule expression in this model.

## Methods

### Study participants

Umbilical cord vein samples were obtained after uncomplicated term elective caesarean section deliveries under regional anaesthesia at University College London Hospital (Grafton Way, London). Written informed consent was obtained from all patients prior to their enrolment in the study.

Adult peripheral blood and AECs were obtained from healthy donors at UCL Great Ormond Street Institute of Child Health. Written informed consent was obtained from all donors prior to their enrolment in the study. Donor characteristics are described in table 1.

**Table 1:** Characteristics of study participants

Variable	Infant cord blood	Adult blood
Donors (*n*)	17	15
% Male	47	26.67
Age (y) (mean (range))	0	29.5 (19–40)
Maternal age (y) (mean (range))	35.8 (range 26–42)	n/a
Gestational age (wk)	38.93 (range 37.9–40.9)	n/a
Neutrophil characteristics		
Blood draw volume	12 mL (5–20 mL)	50 mL
Neutrophil count (cells/mL blood)	2.88×10^6^ (1.83×10^6^)	1.9×10^6^ (2.82×10^6^)
Baseline CD11b (mean fluorescence intensity)	1.52×10^5^	1.39×10^3^
Baseline CD64 (mean fluorescence intensity)	3.49×10^6^	3.38×10^4^
Elastase (mean fluorescence intensity)	6.72×10^5^	1.44×10^4^
MPO (mean fluorescence intensity)	6.04×10^5^	2.17×10^4^

MPO, myeloperoxidase.

### Blood sample collection and neutrophil preparation

Venous and umbilical cord blood was collected by syringe into K+EDTA (Potassium EDTA) tubes (Greiner). Neutrophils were then ultrapurified using an EasySep Direct Neutrophil isolation kit (Stem Cell Technologies) according to the manufacturer’s instructions and processed as described previously.^31^

### AEC culture and neutrophil transepithelial migration model

**Figure 1:**
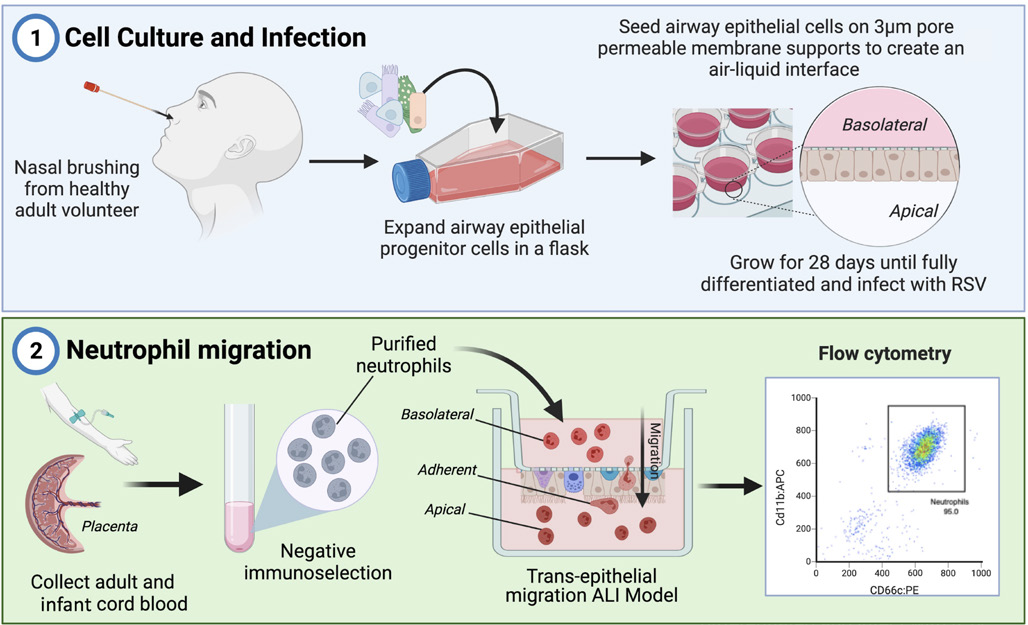
Schematic of experimental protocol including adapted inverted transepithelial migration model. (1—airway epithelial cell (AEC) culture) Primary human AECs were collected from healthy volunteers by nasal brushing, expanded in coculture and seeded onto the underside of PET membranes (Greiner Thincert) as described previously by Herbert *et al*.^15^ These cultures were then grown at air–liquid interface until 28 days, then infected with RSV 72 hours before migration experiments. (2—neutrophil migration) Venous blood from healthy adult volunteers and cord blood from healthy term caesarean deliveries (infant) were collected, neutrophils were then isolated using negative immunoselection. Purified adult or cord blood neutrophils the characterised using flow cytometry and added to the basolateral side of AEC cultures and allowed to migrate for 1 hour.

This study used the transepithelial migration model described by Herbert *et al* 15 to compare the adult and infant neutrophil migration through differentiated 28-day matured ciliated nasal AEC cultures as described previously.^15^ Culture schematic and experimental protocol are visually represented in figure 1. Adult nasal AECs were cultured on porous PET inserts (Greiner) with pore size of 3 µm, which is permissive to neutrophil migration. AECs were infected apically with RSV 72 hours prior to the addition of neutrophils. Mock-infected AECs (Mock) were used as a control for absence of RSV infection or inflammatory proteins secreted by RSV-infected AECs, and Mock-infected AECs with RSV-infected AEC supernatant (RSV Sup) were used as a control to provide the inflammatory milieu secreted by RSV-infected AECs without RSV infection, as described previously.^15^ 400 µL of supernatant from the respective RSV or Mock-infected AECs was added underneath each membrane insert. Neutrophils were then added to the basolateral side of all membrane inserts and incubated at 37°C. A time point of 1 hour was chosen to ensure epithelial integrity was maintained, as measured per previous studies.^15^ After 1 hour, non-adherent neutrophils were collected in the supernatants from the basolateral and apical side of the epithelial cells. Those that remained adhered to AECs were collected by scraping the membrane into 20 µL of media. Excess supernatant after neutrophil migration was collected and stored at −20°C for quantification of secreted neutrophil products.

### Neutrophil quantification, viability and cellular markers

Recovered neutrophils were identified and quantified by CD11b^+^ (CD11b-APC—Cambridge Bioscience 20-0118 T025) using absolute count in measured volume. Viability, apoptosis and cell death were determined by costaining with Annexin-V-FITC and propidium iodide (PI) as described previously.^32^ Viability was defined as FITC^−^PI^−^, apoptosis as FITC^+^PI^−^ and cell death as PI^+^.

Four markers of neutrophil expression were chosen for analysis. CD11b is an integrin essential for neutrophil migration which has been found in greater amounts on neutrophils from infants with RSV Bronchiolitis.^13^ CD64 is an Fc receptor mediating phagocytosis, also shows greater expression on lung neutrophils in murine models of RSV, and is used as a biomarker of sepsis in neonates.^33 34^ NE and MPO are effector enzymes produced in neutrophil azurophilic granules. Cellular expression of CD11b, CD64, NE and MPO was determined by postmigration staining with antibodies CD11b-FITC (Cambridge bioscience 35-0118 T100), CD64-PE-CY7 (BD, 561191), NE-PE (sc-55549PE), MPO-APC (Miltenyl Biotec, 130-107-177) as described previously.^32^

### Flow cytometry

Flow cytometry was performed using a CytoFLEX S fitted with four lasers: 405 nm, 488 nm, 561 nm and 638 nm, at UCL GOS Institute of Child Health Imaging and Flow Cytometry Facility (20 Guilford Street, London). Analysis and gating were performed using FlowJo V.10 (Treestar).

### Virus purification and quantification

The recombinant GFP tagged RSV A2 strain was kindly provided by Jean-Francois Eleouet and described in Fix *et al*.^35^ Viral stock preparation and quantification of viral titre were performed using HEp-2 cells (ATCC CCL-23) as described previously.^15^

### Statistical analysis

Statistical analysis was performed using GraphPad Prism V.9 and R V.4.1.0.^36^ Linear mixed-effect models including a random effect term in the intercept to adjust for unobserved donors’ variability were fitted to compare neutrophils across populations. We used the lme function in the R package nlme.^37^ Normality in the response variable and the random effects, and heterogeneity of variances were tested using the Shapiro-Wilk and Bartlett’s tests. Individual comparisons were performed with a two-way analysis of variance (ANOVA) with pairing and Geisser-Greenhouse correction. ELISA data were analysed for individual comparisons using one-way ANOVA with Tukey’s adjustment for multiple comparisons. Non-normality and lack of homogeneity of variances were dealt with by log-transforming the response variables.

## Results

### Infant neutrophils are smaller and more activated than adult neutrophils at baseline

**Figure 2:**
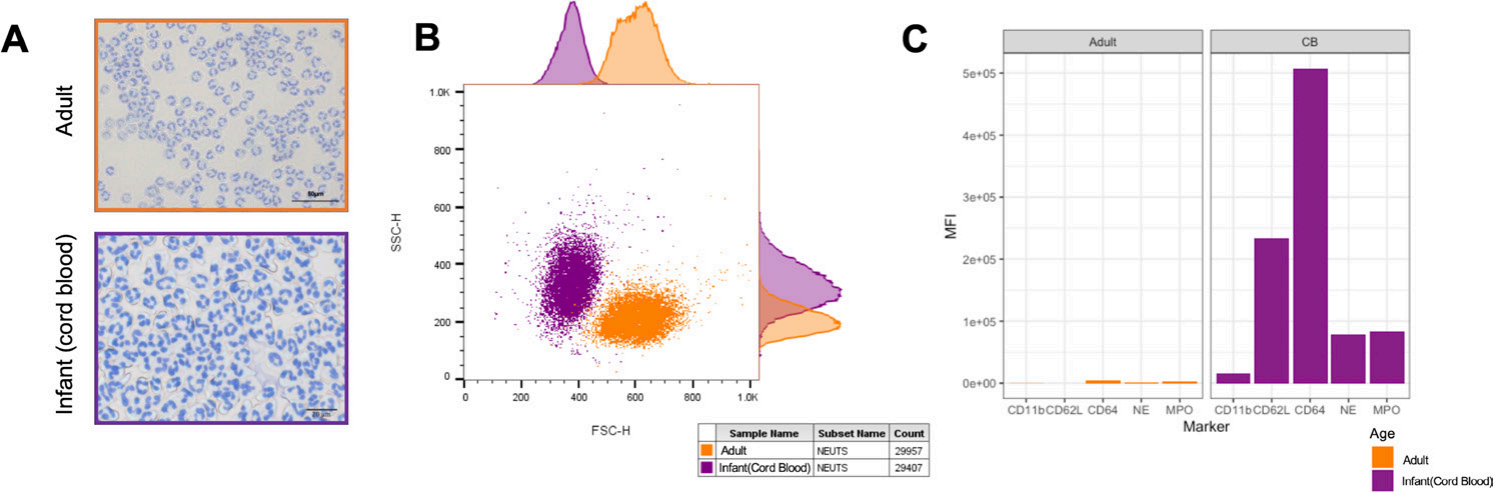
Baseline size, density, viability and expression of key functional markers of infant and adult neutrophils. (A) Purified populations of adult and infant (cord blood) neutrophils cytospun onto a glass slide and stained with Diff-QuikTM. (B) Flow cytometry plot showing forward scatter on the x-axis (size) and side scatter on the y-axis (density) of isolated unstimulated pure populations of infant (purple) and adult (orange) neutrophils. (C) Mean fluorescence intensity per cell of CD11b, CD64, NE, MPO expression on adult (orange) and infant (purple) neutrophils at baseline.

First, we compared the size, density, baseline viability and expression of key functional markers of infant (cord blood) and adult neutrophils. We found that, as a trend, infant neutrophils were smaller than adult neutrophils (FSC) and showed greater variability of size and density (SSC) (figure 2A—representative microscopy, figure 2B—representative plot). The departures from normality and homoscedasticity were significant even after log_10_ transformation of the response variable. However, we report the results from the models’ results in the original scale for clarity and because due to the small sample size we judged that such departures did not impair the analyses’ statistical power. There was no significant difference between the proportions of viable infant (mean±SE (SEM)) (99.67%±0.37) and adult neutrophils (99.55%±0.126). Similarly, we found no significant differences in proportions of apoptotic or unviable infant and adult neutrophils. However, infant neutrophil expression (mean fluorescence intensity (MFI)) of CD11b (15401±393), NE (61 912±9076), MPO (48 752±1863), CD64 (299 705±131 098) and CD62L (170 164±31 113) was significantly greater than adult neutrophils, CD11b (147±25), NE (1524±95), MPO (2616±535), CD64 (3383±649) and CD62L (241±173), respectively (p<0.0001 for all comparisons, figure 2C).

### Greater total numbers of infant neutrophils migrate across and remain adherent to RSV-infected AECs in comparison to adult neutrophils

Next, we compared the propensity of infant and adult neutrophils to migrate across RSV-infected AECs, by counting the total number of neutrophils in three compartments of our in-vitro human airway model: basolateral, migrated or adherent to AECs (figure 1) after 1-hour incubation.

#### Infant neutrophils

We found that RSV infection resulted in greater numbers of infant neutrophils recovered apically with 336 684±48 129 neutrophils migrated across RSV-infected AECs (p=0.024) and RSV sup (236 870±3359) (p=0.011) in comparison to Mock-infected AECs (96 268±2859). Reciprocally, we found fewer infant neutrophils were recovered basolateral to RSV-infected AECs (25 902±5715) (p=0.04) and RSV sup (16 428±1171) (p=0.012) in comparison to Mock-infected AECs (190 158±32 008). We found no significant difference in numbers of infant neutrophils adhered to Mock, RSV Sup or RSV-infected AECs.

#### Adult neutrophils

We found no significant difference in numbers of adult neutrophils recovered apically or adherent to Mock, RSV sup or RSV-infected AECs. However, we found significantly fewer adult neutrophils were recovered basolateral to RSV-infected AECs (124 411±5715) in comparison to RSV Sup AECs (311 142±23 975)(p=0.03).

**Figure 3:**
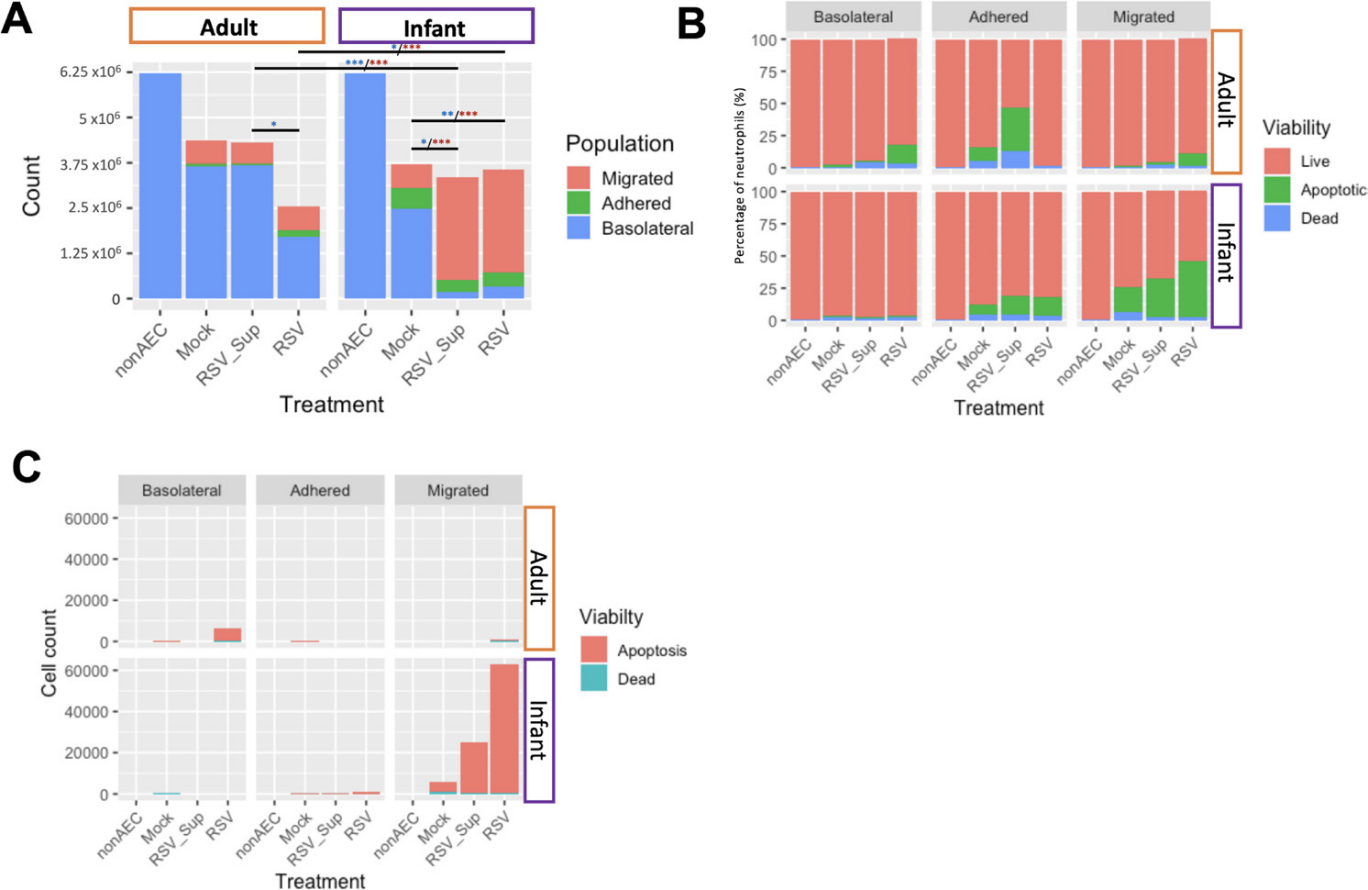
Greater numbers of infant neutrophils migrate across respiratory syncytial virus (RSV)-infected airway epithelial cells (AECs) in comparison to adult neutrophils. Neutrophils isolated from healthy adult venous blood or cord blood (infant) from healthy infants born at term were added to the basolateral side of ciliated adult AEC cultures infected with RSV for 72 hours. After 1-hour migration, neutrophils on the basolateral and apical sides of AECs, and those adhered to the AECs were counted by flow cytometry. (A) Total numbers of neutrophils in stacked bars isolated from the basolateral (blue), adhered (green) and apical (red) compartments of the culture, as illustrated on the schematic (figure 1). Blue stars indicate differences found between basolateral counts, yellow stars indicate differences between adhered counts burgundy stars indicate differences found between apical counts. (B) Proportions of neutrophil viability, apoptosis and cell death. Neutrophils recovered from the basolateral to AECs, adhered to AECs, and apical to AECs are shown. (C) Absolute counts of neutrophils which were apoptotic or dead. Neutrophils recovered from the basolateral to AECs, adhered to AECs, and apical to AECs are shown. Both differential counts and relative proportions of neutrophils which were live (red), apoptotic (green) and dead (blue) after 1-hour incubation were analysed using two-way analysis of variance with Greenhouse-Geisser post hoc test for multiple comparisons. *P<0.05, **p<0.002, ***p<0.0001.

Comparing infant and adult neutrophils, we found greater numbers of infant neutrophils (336 684±48 129) migrated to the apical compartment of RSV-infected AECs in comparison to adult neutrophils (56 586±16 139) (p<0.0001) (figure 3A). This corresponded with greater numbers of adult neutrophils (124 411±5715) remaining basolateral to RSV-infected AECs than infant neutrophils (25 902±5715) (p=0.0004). There was no significant difference between adult and infant neutrophils remaining basolateral to Mock-infected AECs. Greater numbers of infant neutrophils remained adherent to Mock-infected AECs (45 440±6573) and Mock-infected AECs with RSV-infected AEC supernatant placed apically (RSV Sup) than adult neutrophils (4673±2319) (p=0.038). There was no significant difference in numbers of infant (41 972±3832) and adult neutrophils (15 065±2388) adherent to RSV-infected AECs.

### A greater proportion of infant neutrophils are apoptotic following transepithelial migration

To compare the viability of infant and adult neutrophils following transepithelial migration, we measured the proportion of viable, dead or apoptotic neutrophils at single cell level using flow cytometry. As a control condition, we exposed matched neutrophils to media alone for 1 hour (NonAEC). Data are summarised as mean%±SEM.

We found that fewer infant neutrophils remained viable following migration across Mock (74.6% compared with 98.5% adult) and RSV-infected AECs (54.3% to 89.6%) (figure 3B). Examining the neutrophils which migrated across RSV-infected AECs, we found that a significantly greater percentage of infant neutrophils (43.3%±10.68) underwent apoptosis compared with adult neutrophils (9.49%±2.7) (p=0.0024) (figure 3B). Similarly, we found more apoptotic infant neutrophils recovered *apically* to RSV Sup AEC cultures (30.0%±10.01%) in comparison to adult neutrophils (2.48%±2.11) (p=0.014).

Examining the adult and infant neutrophils which remained *adhered* to RSV-infected AECs, we found that infant neutrophils (14.6%±1.8) had significantly greater proportion of apoptosis in comparison to adult neutrophils (0.803%±0.31) (p=0.0192) (figure 3B). Conversely, adult neutrophils *adhered* to RSV Sup AEC cultures (33.6%±7.4) showed a significantly greater proportion apoptotic in comparison to infant neutrophils (14.4%±2.26) (p=*0.0198*).

We did not find a significant difference in proportion of apoptosis between infant and adult neutrophils recovered basolateral to Mock-infected AECs and RSV Sup AECs.

Counting absolute numbers of apoptotic neutrophils (as opposed to proportions presented above), we found greater numbers of apoptotic infant neutrophils (140 787±12 199) were recovered *apically* after migration across RSV-infected AECs in comparison to adult (5853±2759) (figure 3C) (p=0.002). Similarly, there were greater numbers of apoptotic infant neutrophils (5566±698) *adhered* to RSV-infected AECs in comparison to apoptotic adult neutrophils (111.3±32) (p=0.0001) (figure 3C). We also found greater numbers of apoptotic infant neutrophils *adhered* to Mock-infected AECs (4031±543) in comparison to apoptotic adult neutrophils (111±82) (p=0.002) (figure 3C).

Conversely, we found greater numbers of apoptotic adult neutrophils (29 065±16 101) *basolateral* to RSV-infected AECs in comparison to apoptotic infant neutrophils (406±6.53)(p=0.0261) (figure 3C).

### RSV-infected AECs increases CD11b expression on adult but not infant neutrophils

We then used flow cytometry to quantify, at a single cell level, expression of key cellular markers CD11b and CD64, and secretory effector molecules NE and MPO on infant and adult neutrophils following transepithelial migration. Data are summarised as MFI/cell±SEM.

#### CD11b

**Figure 4:**
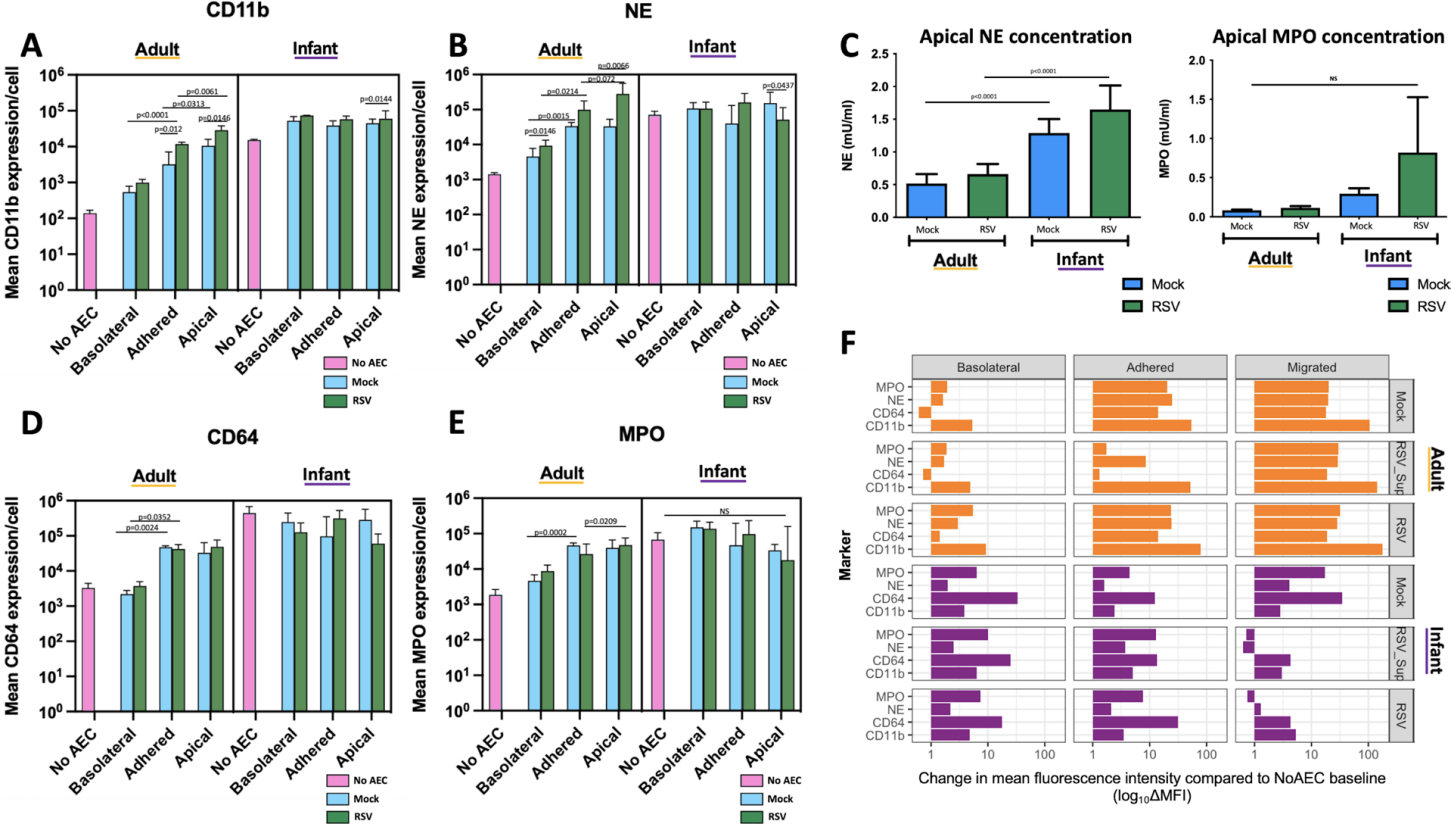
Respiratory syncytial virus (RSV)-infected airway epithelial cells (AECs) increases CD11b expression on adult but not infant neutrophils. (A, B, D and E) Mean expression per cell of CD11b (A), neutrophil elastase (NE) (B), CD64 (D), myeloperoxidase (MPO) (E) on adult (left—orange) and infant (cord blood) (right—purple) neutrophils recovered from the basolateral, adhered and apical compartments of transepithelial migration assays across Mock (blue), and RSV (green)-infected AECs. Media only controls are shown in pink. Mean fluorescence intensity (MFI) of each marker measured is shown on a log10 scale. (C) Concentrations of NE (left) and MPO (right) in the apical supernatants collected after neutrophil migration across ciliated AECs infected with RSV for 72 hours (RSV), Mock-infected control AECs infected for 72 hours (Mock). Concentrations determined by ELISA. Individual comparisons performed using one-way analysis of variance (ANOVA) with Tukey’s adjustment for multiple comparisons. (F) Difference in MFI in comparison to media-only control (NoAEC) expressed as log10 per cell of CD11b, CD64, NE, MPO on adult (upper—orange) and infant (cord blood) (lower—purple) neutrophils recovered from the basolateral, adhered and apical compartments of transepithelial migration assays across Mock, RSV Sup and RSV-infected AECs. A linear mixed effects model was used to compare interactions between groups and control for donor variability. Individual comparisons performed with two-way ANOVA with pairing and Geiser greenhouse correction and not shown on graph.

We found no significant difference in CD11b expression between infant neutrophils recovered basolateral, adherent to or apical to RSV-infected AECs (figure 4A). Adult neutrophils recovered adherent to RSV-infected AECs (10,995±562.1) exhibited greater expression of CD11b compared with those recovered basolateral to AECs (1,206.67±178.6) (p<0.0001) and adult neutrophils recovered apically (28 587±3853) showed greater expression of CD11b compared with those adhered to RSV-infected AECs (p=0.0061) (figure 4A). Infant neutrophils recovered basolateral to RSV-infected AECs (73 140±896.3) showed greater CD11b expression than adult neutrophils AECs (1206.67±178.6) in the same conditions (p<0.0001). Similarly, infant neutrophils adhered to RSV-infected AECs (53 561±8186) showed greater expression of CD11b in comparison to adult neutrophils (10 995±563) adhered to RSV-infected AECs (p=0.00065). Infant neutrophils recovered apical to RSV-infected AECs (71 152.7±196 790.6) showed greater CD11b expression than adult neutrophils (28 587.8±3853.4) (p=0.05) (figure 4A).

Overall, in all compartments, infant neutrophils showed considerably greater CD11b expression than adults as compared by linear mixed effects model (p<0.0001). Additionally, while there was no noticeable rise in CD11b expression as infant neutrophils moved from the basolateral to adhered to apical compartments (unlike in adult neutrophils), the baseline CD11b expression in infants was still higher than that observed in adult neutrophils at any stage of migration.

#### Neutrophil elastase

There was no significant difference in NE expression between infant neutrophils recovered basolateral, adherent to or apical to RSV-infected AECs. Adult neutrophils recovered adherent to RSV-infected AECs (36 709±2147) exhibited greater expression of NE compared with those recovered basolateral to AECs (4680±1362) (p=0.0214) and adult neutrophils recovered apically (43 164.67±1053.8) showed greater expression of NE compared with those adhered to RSV-infected AECs (p=0.0066) (figure 4B). Comparing media-only controls between adult and infant neutrophils, mean NE expression was 61 912 (±9076) on infant neutrophils and 1524 (±95) on adult neutrophils (figure 4B).

Examining secreted NE concentration in apical AEC supernatant, greater concentrations of NE were present after infant neutrophil transepithelial migration (1.65±0.37 mU/mL) in comparison to adult neutrophil migration (0.661±0.15mU/ml)(p<0.0001)(figure 4C).

#### CD64

There was no significant difference in CD64 expression between infant neutrophils recovered basolateral, adherent to or apical to RSV-infected AECs. CD64 expression on adult neutrophils adhered to RSV-infected AECs (43 103±80 650.9) showed greater expression than those found basolateral (4429.7±710.2). Comparing media-only controls between adult and infant neutrophils, mean CD64 expression was 349 029 (±11785) on infant neutrophils and 3383 (±649) on adult neutrophils (figure 4D).

#### Myeloperoxidase

Adult neutrophils recovered apical to RSV-infected AECs (53 559.67±11 269) exhibited greater expression of MPO compared with those recovered adhered to AECs (31 333.3±10 036) (p=0.0209) (figure 4E). We saw no significant difference in apical supernatant MPO concentration after infant neutrophil transepithelial migration in comparison to adult neutrophil migration (figure 4C).

#### Fold change comparison

As described earlier, infant neutrophils displayed higher baseline expression of cellular markers compared with adult neutrophils (figure 2C). To further interpret the response of neutrophils to RSV-infected AECs, the difference in expression of NE, MPO, CD64 and CD11b from baseline (No AEC) was calculated and expressed as log_10_ change (figure 4F). Examining neutrophils basolateral to AECs, there was no significant difference in change of NE, MPO, CD64 and CD11b from baseline between adult and infant neutrophils. However, on examining the change in expression of neutrophils adhered to RSV-infected AECs, adult neutrophils showed greater expression of NE (23.1±1.22), MPO (22.5±9.33), CD64 (13.11±3.84) and CD11b (76.97±9.62) relative to adult No AEC control than was seen on infant neutrophils NE (1.09±0.899), MPO (6.69±4.88), CD64 (30.25±30.66) and CD11b (2.46±0.44) relative to infant No AEC(figure 4F). Apically, adult neutrophils showed greater difference in expression of NE (27.4±1.18), MPO (30.84±16.32), CD64 (17.9±5.70) and CD11b (174.3±29.5) compared with no AEC than was seen with infant neutrophils NE (0.29±0.36), MPO (−0.25±0.5), CD64 (3.23±4.01) and CD11b (4.28±1.42) compared with no AEC respectively.

## Discussion

This study has shown, for the first time, differences in the number and viability of infant (term cord blood) and adult neutrophils in an in-vitro model of RSV bronchiolitis, and associated differences in neutrophil activation marker expression.

First, we found significantly greater numbers of infant neutrophils that migrated to the apical side of RSV-infected AECs compared with adult neutrophils. This finding is in keeping with clinical studies of infants with severe RSV bronchiolitis which identified abundant neutrophil infiltrate in the infant lung and provides first evidence that this may be a distinct phenomenon which is not a feature of the host response to airway infection in adults.^10^ Human adult challenge models of RSV infection have correlated pre-existing neutrophilic inflammation of the upper airway mucosa with worsened symptomatic infection.^19^ This may suggest that pre-existing neutrophil infiltration predisposes to a neutrophil dominant inflammation during subsequent RSV infection and associated with more severe symptoms. A recent study examining the immune profiles of children requiring treatment as either an inpatient or an outpatient with RSV showed that robust innate interferon responses to RSV infection were correlated with mild disease, whereas increased neutrophil-linked and inflammatory genes were correlated with more severe disease.^38^ Further clinical studies are required to further elucidate this relationship; however, in separate work, we hypothesise neutrophil chemotaxis towards airway lumen is amplified by a single dying neutrophil, a phenomenon that has been evidenced previously in models of sterile injury.^39 40^ We recovered significantly fewer adult neutrophils after migration across RSV-infected AECs than in other experimental conditions, which could be attributed to either a biological or an experimental process and is yet unclear. It is possible that adult neutrophils are being removed from the system, by biological process such as apoptosis, more quickly than neonatal neutrophils under the same conditions and this is relevant to resolution of inflammation.

Second, we found that a greater proportion of migrated (apical) infant neutrophils were apoptotic when exposed to RSV-infected AECs, compared with adult neutrophils where the majority were viable, except for adult neutrophils basolateral to RSV-infected AECs. This was an unexpected finding since clinical evidence suggests neutrophils from the BAL of infants with RSV exhibit prolonged viability, but the mechanisms have not yet been defined and it may be that these neutrophils, recovered in the BAL, are not typical of all neutrophils in the RSV-infected airways of babies.^41^ In clinical studies, it has been shown that infant airways secretions, both with and without RSV infection, have an anti-apoptotic effect on adult neutrophils in-vitro in the absence of airway.^41^ This was not true for adult airway secretions. Few studies have directly measured the viability, apoptosis and cell death of neutrophils recovered from infants. One such study, in support of our findings, showed increased apoptosis in neutrophils recovered from nasopharyngeal aspirate compared with bloodstream neutrophils in children with RSV.^42^ It is possible that the large apical infiltration of infant neutrophils and large proportion of apoptosis may be a protective mechanism; the neutrophil infiltrate may combat the viral infection, with appropriate apoptosis of obsolete cells to prevent inflammatory cell death and abrogate ongoing inflammation. Disease severity may be a result in a failure of this process to control inflammation, or the combination of heightened inflammation with physiologically smaller airways prone to plugging. Interestingly, the proportion of adherent neutrophils which were apoptotic was significantly greater in adult neutrophils exposed to RSV Sup than those exposed to RSV-infected AECs themselves, which suggested that the presence of virally infected cells as opposed to the inflammatory milieu may be responsible.

To further investigate neutrophil function during transepithelial migration in response to RSV infection, we examined neutrophil markers correlated with key functions such as migration (CD11b), pathogen recognition (CD64), phagocytosis and degranulation (NE and MPO). CD11b is an integrin known to facilitate transepithelial migration in-vivo. The blood of infants with RSV bronchiolitis has been shown to contain neutrophils with greater neutrophil CD11b expression compared with uninfected children.^13^ This study also demonstrated greater CD11b expression on neutrophils in the BAL in comparison to the blood of infants with RSV bronchiolitis. Our in-vitro model results consistently mirrored these clinical observations. Specifically, we noted that infant neutrophils demonstrated constitutively greater CD11b expression at baseline in comparison to adult neutrophils. Furthermore, there was limited capacity for infant neutrophils to further upregulate this in response to RSV infection and/or migration, in contrast to adult neutrophils which demonstrated a higher degree of upregulation in similar conditions. However, even with this upregulation, the final expression level in adult neutrophils remained considerably lower than that observed in infant neutrophils, even at baseline.

There is limited research into CD11b on adult neutrophils during RSV infection in vivo. Sequestration of primed neutrophils in the lungs, as separate to the general circulation, is a key protective mechanism shown to be dysregulated in acute respiratory distress syndrome (ARDS), a similar clinical syndrome to severe bronchiolitis.^43^ CD11b has been found to be raised on blood and BAL neutrophils in patients with ARDS secondary to infection compared with healthy volunteers.^44^ In vitro, we have demonstrated that adult neutrophils upregulate CD11b in response to RSV and in response to migration across RSV-infected AECs,^15 31 32^ suggesting a potential similarity in underlying pathology.

This study had several limitations, which can be addressed in future studies. Our in-vitro model does not contain other immune cells, which may influence the behaviour and responses of neutrophils in-vivo during RSV infection.^45 46^ We did not measure other neutrophil functions, such as NET formation, azurophilic granule translocation or generation of reactive oxygen species in our model, due to the scarcity of sample and small scale of experiments, which may contribute to the observed results in our study and may be a direction of future work. In addition, due to their availability, our airway model was prepared from adult nasal AECs whereas RSV causes a severe bronchiolitis in infants, so a bronchially derived brushing sample may be a more clinically valid reflection. Nasal AECs have been used previously in in-vitro models of RSV infection due to their reproducibility and permissibility to in-vitro infection with RSV.^15^ It would also be preferrable to use infant AEC samples to ensure a truer representative model; however, obtaining these samples from infants is prohibitively difficult for technical and ethical reasons, and although commercial suppliers may offer suitable cells, proprietary preparation and preservation techniques may limit utility and comparability to primary donors. This study employed linear mixed-effect models to assess group interactions while controlling for donor variability. However, despite log10 transformation of the response variable, deviations from normality and homoscedasticity remained, which is of important consideration when interpreting the findings.

Similarly, as has been done previously,^21 28–30^ this study used neutrophils isolated from cord blood as a surrogate for infant neutrophils. In this study, it would not have been possible logistically or ethically to collect adequate volume of blood from infants. Cord blood neutrophils are isolated immediately after birth and transition from a relatively hypoxic and sterile environment which may lead to differences in neutrophil gene expression, cell surface marker expression and functionality which are not yet defined. Two small studies have suggested that cord blood neutrophils after vaginal delivery, compared with after caesarean section, show greater chemotactic ability, IL-8 secretion and responsiveness to stimulation^47 48^ and so we collected cord blood following elective term caesarean births.

## Concluding remarks

We showed greater numbers of infant neutrophils migrate across RSV-infected AECs compared with adult neutrophils, a greater proportion of which remained viable after migration. Infant neutrophils showed constitutively greater expression of CD11b which may facilitate migration. We also showed several factors contribute to increased inflammation on the apical side of AECs including increased concentration of NE, and greater proportions of infant neutrophils migration. These findings provide further context to previous clinical observations, suggesting that recruitment of neutrophils to RSV-infected airways may contribute to clinical severity in infants with RSV bronchiolitis.

## Data Availability

All data relevant to the study are included in the article or uploaded as supplementary information.
